# Cardiorespiratory adaptations in small cetaceans and marine mammals

**DOI:** 10.1113/EP091095

**Published:** 2023-11-15

**Authors:** Andreas Fahlman

**Affiliations:** ^1^ Global Diving Research SL Valencia Spain; ^2^ Fundación Oceanogràfic de la Comunidad Valenciana Valencia Spain; ^3^ Kolmården Wildlife Park Kolmården Sweden; ^4^ IFM Linköping University Linköping Sweden

**Keywords:** cetacean, diving physiology, heart rate, marine mammal, perfusion

## Abstract

The dive response, or the ‘master switch of life’, is probably the most studied physiological trait in marine mammals and is thought to conserve the available O_2_ for the heart and brain. Although generally thought to be an autonomic reflex, several studies indicate that the cardiovascular changes during diving are anticipatory and can be conditioned. The respiratory adaptations, where the aquatic breathing pattern resembles intermittent breathing in land mammals, with expiratory flow exceeding 160 litres s^−1^ has been measured in cetaceans, and where exposure to extreme pressures results in alveolar collapse (atelectasis) and recruitment upon ascent. Cardiorespiratory coupling, where breathing results in changes in heart rate, has been proposed to improve gas exchange. Cardiorespiratory coupling has also been reported in marine mammals, and in the bottlenose dolphin, where it alters both heart rate and stroke volume. When accounting for this respiratory dependence on cardiac function, several studies have reported an absence of a diving‐related bradycardia except during dives that exceed the duration that is fuelled by aerobic metabolism. This review summarizes what is known about the respiratory physiology in marine mammals, with a special focus on cetaceans. The cardiorespiratory coupling is reviewed, and the *selective gas exchange* hypothesis is summarized, which provides a testable mechanism for how breath‐hold diving vertebrates may actively prevent uptake of N_2_ during routine dives, and how stress results in failure of this mechanism, which results in diving‐related gas emboli.

## Abstract (highlights)

- **What is the topic of this review?**
  Current knowledge of the respiratory physiology in small cetaceans and its influence on cardiac function.
- **What advances does it highlight?**
  The selective gas exchange hypothesis is presented, which is a framework for how marine mammals manage gases during diving and is based upon the current understanding of cardiorespiratory coupling in breath‐hold diving vertebrates.

## 1 INTRODUCTION

Marine mammals have fascinated physiologists for decades due to their dependence on the surface for breathing, while their food is located underwater. The aquatic environment represents numerous physiological challenges for homeotherms. For example, the higher thermal conductivity of water as compared with air makes thermoregulation a challenge. For breath‐hold diving homeotherms, the increasing hydrostatic pressure with depth and the absence of air are additional challenges, resulting in alveolar compression, hypoxia and hypercapnia. The pressure‐dependent pulmonary shunt that develops during descent, and lack of access to oxygen must be managed to avoid trauma during diving. Marine mammals have a respiratory system specifically designed for efficient function in air and water, with efficient cardiorespiratory coupling that enhances gas exchange and recovery during brief periods at the surface (Fahlman et al., 2017; Ponganis, 2015). A large proportion of the studies on diving physiology have focused on the management of the available O_2_, by estimating or measuring the metabolic cost of diving and the available O_2_ stores. Another area of interest has been the cardiovascular changes associated with diving, or what Scholander (1963) called the ‘master switch of life’. The dive response has been proposed to be a reflex adaptation for diving (Scholander, 1963), but a number of studies have shown that the response is highly variable and can be conditioned (Elmegaard et al., 2016; Elsner et al., 1966; Fahlman, Cozzi, et al., 2020; Lin et al., 1972; Ridgway et al., 1975). As both terrestrial and aquatic vertebrates respond with a deceleration in heart rate when submerged, some have questioned that this response specifically evolved as an adaption to diving, but is an ancestral response against hypoxia (Mottishaw et al., 1999).

Although the lungs are vital for gas exchange and assure delivery of O_2_ and removal of CO_2_, the understanding of the respiratory physiology of diving mammals is limited. One of the hallmarks of the respiratory physiology of marine mammals is the so‐called aquatic breathing strategy (Fahlman et al., 2017; Ridgway, 1972), or what is also called intermittent breathing (Milsom et al., 2022). While intermittent breathing is common in reptiles, amphibians and air‐breathing fish, it is limited in terrestrial mammals, but can occur at regular intervals in, for example, hibernating mammals (Milsom et al., 2022). However, the intermittent breathing in hibernating squirrels is very different from the aquatic breathing strategy of most aquatic mammals, where the respiratory pause in the former begins following exhalation while in the latter it begins following inhalation (Fahlman et al., 2017; Milsom, 1991; Milsom et al., 2022). Thus, the aquatic breathing strategy is like intermittent breathing in amphibians and reptiles, but different as compared with terrestrial mammals, and therefore deserves its own classification.

In the following review, the aim is to describe what is known about the aquatic breathing strategy in marine mammals with a specific focus on cetaceans. The review includes work to assess the functional characteristics (compliance) of the respiratory system, lung inflation experiments on cadavers and anaesthetized animals, and lung function studies on awake animals in professional care. The review details the cardiorespiratory coupling where respiration alters cardiac function, as it may be a key mechanism that improves gas exchange while reducing cardiac work. Finally, both empirical and theoretical studies on the cardiorespiratory physiology in cetaceans have resulted in the *selective gas exchange* hypothesis. This hypothesis is reviewed as a potential mechanism for how these animals manage gases during diving and how they avoid diving‐related issues like decompression sickness (DCS), that is, the bends.

### 1.1 Respiratory physiology in marine mammals: the Scholander legacy

In air breathing mammals, the lungs provide the blood with an exchange surface that allows exchange of O_2_, CO_2_ and N_2_. The rate of gas exchange depends on the diffusion rate, which in turn depends on the total alveolar surface area in contact with blood, the partial pressure gradient, the diffusion distance and diffusion constant, that is, Fick's law of diffusion. During a breath, the air brought in mixes with the gas in the lung, which is low in O_2_ and high in CO_2_. The new concentration of gases following inhalation depends on the relative volume brought in (tidal volume), the dead space volume (the conducting airways) and the volume and concentration of gas left in the lung following exhalation. Following inhalation, the partial pressures of O_2_ and CO_2_ are increased and decreased, respectively, which helps increase the diffusion gradient of O_2_ and CO_2_. In continuous breathers at rest, the alveolar partial pressures of O_2_ ($P_{O_{2}}$) and CO_2_ ($P_{{\mathrm{CO}}_{2}}$) are therefore relatively constant. In intermittently breathing marine mammals, and especially in cetaceans, the alveolar (end‐tidal) partial pressures vary substantially, but the $P_{O_{2}}$ is generally lower and the $P_{{\mathrm{CO}}_{2}}$ higher as compared with terrestrial mammals (Fahlman et al., 2015; Fahlman, Rhieu, et al., 2023), although there are studies that report end‐tidal $P_{{\mathrm{CO}}_{2}}$ similar to terrestrial mammals (Mortola & Limoges, 2006).

During diving, the lung contains a finite supply of O_2_ that can act as a reservoir to help fuel aerobic metabolism, but the increasing pressure causes compression of the gas‐filled alveolar space, which results in pressure‐dependent changes in the diffusion rate, that is, a physiological pulmonary shunt (Bostrom et al., 2008; Fahlman et al., 2009; Kooyman & Sinnett, 1982; McDonald & Ponganis, 2012). The alveolar compression initially increases the gas diffusion rate as the pulmonary partial pressure increases. As the pressure increases, continued compression of the alveolar space results in reduced surface area for gas exchange, and a longer diffusion distance. This reduces the rate of gas diffusion and increases the pulmonary shunt (Bostrom et al., 2008; Kooyman & Sinnett, 1982). The diffusion rate continues to decrease until complete alveolar collapse (atelectasis) at which time gas exchange ceases (Fahlman et al., 2009; McDonald & Ponganis, 2012). Consequently, alveolar compression will affect the ability to utilize this source of O_2_, but it also helps prevent uptake of N_2_, which reduces the risk for gas emboli to form during ascent (decompression) (Scholander, 1940). This effect of pressure on gas exchange is therefore important to understand how breath‐hold divers manage gases while underwater, and to what extent the lungs can be used as a store for O_2_ and a reservoir for CO_2_ (Fahlman et al., 2009; Fahlman, Sato, et al., 2020).

In 1940, Per Scholander published his seminal work on the respiratory function in diving mammals and birds. He described the anatomy of the lungs as a system made up of a compliant chest and alveoli, connected to stiff conducting airways (Scholander, 1940). This conceptual system was later coined the *balloon‐pipe* model (Bostrom et al., 2008). This simplified model allowed the alveolar collapse depth to be estimated based on the relative volumes of the diving lung and dead‐space volumes using Boyle's law (Scholander, 1940). Modified versions of this model, where the structural properties of the conducting airways and alveolar space, and the relative size of the dead space and diving lung volumes, allowed the pulmonary shunt to be estimated at different depths (Bostrom et al., 2008; Fahlman et al., 2009). These later studies provided theoretical evidence that the alveolar collapse depth is deeper than 100 m, which was later empirically confirmed (Fahlman et al., 2009; McDonald & Ponganis, 2012). However, the alveolar collapse depth based solely on the passive compression of the respiratory system varies with the diving lung volume (Bostrom et al., 2008; Scholander, 1940). As cetaceans and sea lions generally dive following inspiration, this would affect alveolar compression in a way that gas exchange would continue to higher pressures. To better assess these changes, the structural and functional properties of the respiratory system are necessary.

### 1.2 Estimated total lung capacity

Although the total lung capacity (TLC) is difficult to reliably assess, one indirect definition used is the volume required to inflate the respiratory system, that is, excised lung, to a trans‐pulmonary pressure of 30 cmH_2_O (see discussion by Leith, 1976). Based on data from several studies, the estimated TLC (TLC_est_) appears similar between terrestrial and marine mammals. The TLC_est_ for terrestrial mammals is reported as: TLC_est_ (ml) = 53.5 *M*_b_^1.06^ (Stahl, 1967), where *M*_b_ is the body mass in kg. In marine mammals, including large baleen whales and the deep diving pilot whale, TLC_est_ (litres) was 0.135 *M*_b_^0.92^ (Fahlman et al., 2017; Kooyman, 1973). However, based on measurements of excised lungs and anatomical lung size, it has been proposed that the TLC is significantly smaller in some deep diving cetaceans such as the false pygmy sperm whale (*Kogia breviceps*) and members of the ziphiid family, for example, northern bottlenose whale (*Hyperoodon ampullatus*) (Scholander, 1940). One possible explanation is that the lung is not used as an active store of O_2_, and that a smaller lung reduces the likelihood for excessive uptake of N_2_ and formation of gas emboli upon ascent (Scholander, 1940). The smaller lung would therefore cause alveolar collapse at a shallower depth and protect against the risk of gas emboli.

### 1.3 Breathing frequency and tidal volume

**FIGURE 1:**
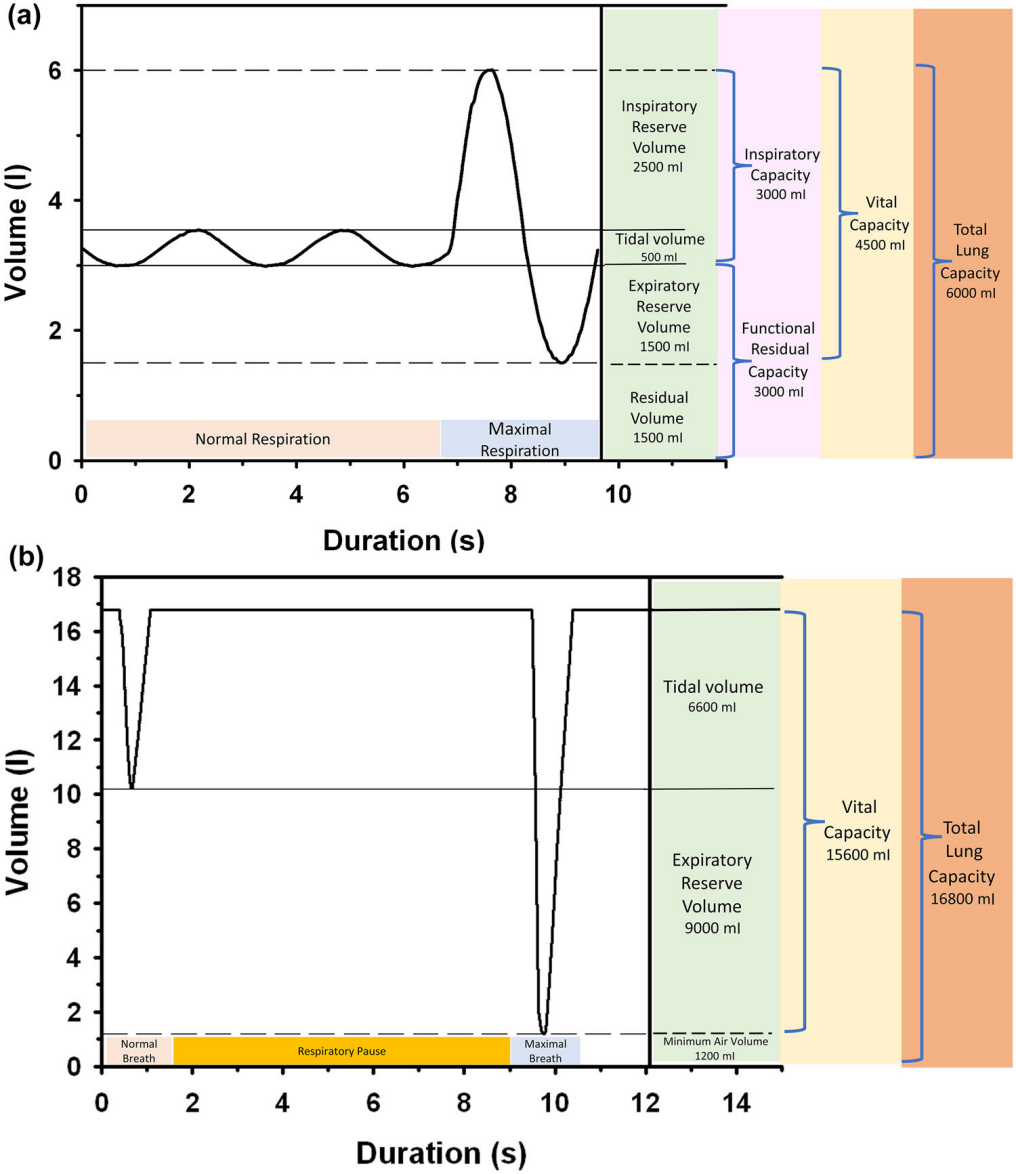
Data showing representative tidal and maximal breaths from 70 kg adult human (a) and 190 kg bottlenose dolphin (b). Figure adapted from Piscitelli, M., Kooyman, G., and Fahlman, A. (2024). Respiratory physiology in dolphins. *The Physiology of Dolphins*, Chapter 5. Elsevier. https://doi.org/10.1016/B978‐0‐323‐90516‐9.00005‐1.

**FIGURE 2:**
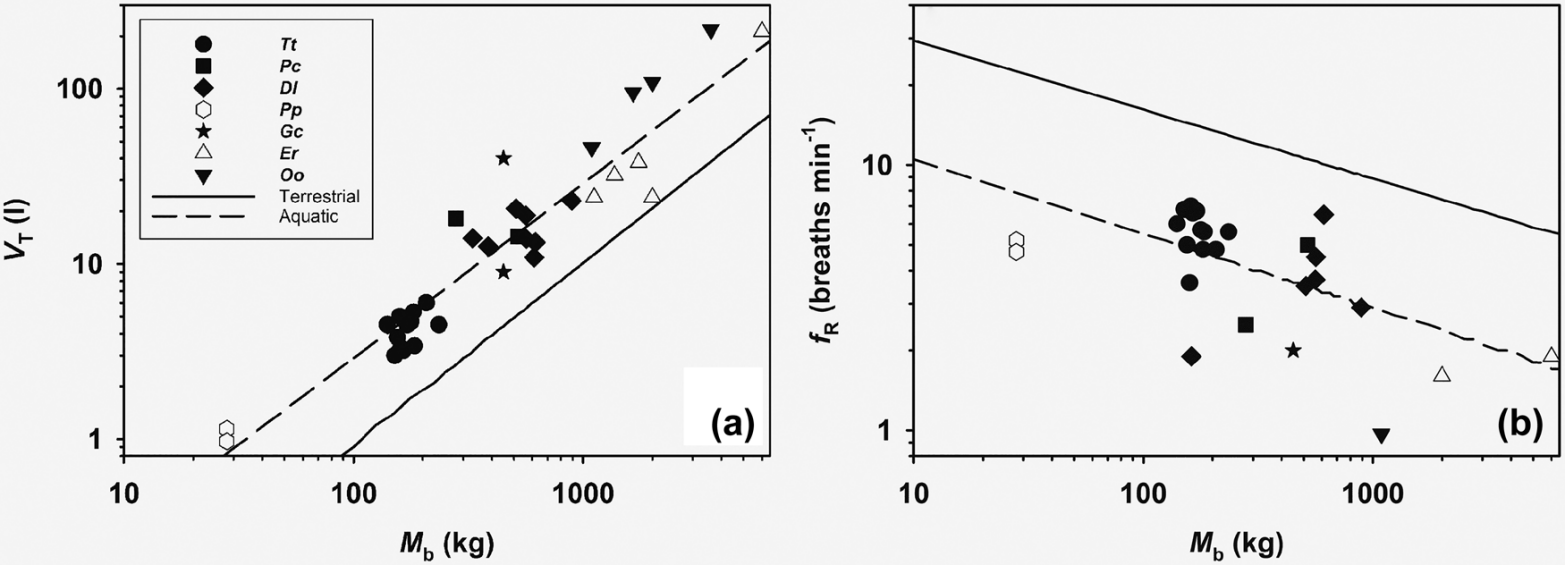
Measured and predicted tidal volume (*V*_T_) (a), and breathing frequency (*f*_R_) (b) during rest in adult bottlenose dolphins (*Tt*, *Tursiops truncatus*), calf and adult beluga (*Dl*, *Delphinapterus leucas*), a juvenile and an adult false killer whale (*Pc*, *Pseudorca crassidens*), adult pilot whale (*Gs*, *Globicephala scammoni*), juvenile harbour porpoises (*Pp*, *Phocoena phocoena*), adult and juvenile killer whale (*Oo*, *Orcinus orca*) and calf grey whales (*Er*, *Eschrichtius robustus*). Continuous and dashed lines are allometric predictions for terrestrial (Stahl, 1967) and aquatic mammals (Fahlman, Borque‐Espinosa, et al., 2020), respectively.

The aquatic breathing strategy is like intermittent breathing in reptiles and amphibians, where the breath begins with an exhalation, followed by inspiration and a respiratory pause (Figure 1) (Fahlman et al., 2017; Milsom et al., 2022). In marine mammals, the tidal volume is higher (Figure 2a), and the breathing frequency is lower (Figure 2b) as compared with terrestrial mammals (Fahlman, Borque‐Espinosa, et al., 2020; He et al., 2023). Allometric scaling is used to describe the relationships between body mass and a physiological variable (He et al., 2023). For example, the relationship between tidal volume and body mass can be described by the following equation: tidal volume = *a* body mass*^b^*, where *a* and *b* are parameters and *b* is often referred to as the allometric mass‐exponent. The allometric relationship between breathing frequency and body mass differs between marine and terrestrial mammals, being −0.25 in terrestrial mammals, and between −0.34 and −0.42 in aquatic mammals (Fahlman, Borque‐Espinosa, et al., 2020; He et al., 2023; Mortola & Limoges, 2006). Although they have slightly different results, these studies agree that the breathing frequency differs between terrestrial and marine mammals, with the latter taking fewer breaths in a similar sized individual.

The tidal volume in marine mammals, on the other hand, is higher in both cetaceans and pinnipeds (Fahlman et al., 2017; He et al., 2023). In the cetacean, the vital capacity can be as high as 80–90% of the total lung capacity (Fahlman et al., 2017, Fahlman, Borque‐Espinosa, et al., 2020), but recent studies in small and medium sized cetaceans have shown that the tidal volume at rest is usually only around 30–40% of the total lung capacity, and increases only slightly even after high intensity exercise or prolonged breath‐holds (Fahlman et al., 2015, 2016; Fahlman, Borque‐Espinosa, et al., 2020). In larger sized cetaceans, the measured tidal volume has been a greater proportion of the total lung capacity, which may reflect species differences or variation in methodology (Kriete, 1995). A number of hypotheses have been proposed for the aquatic breathing strategy, and one is that it provides positive buoyancy in water (Mortola & Limoges, 2006). Another suggestion is that the large lung volume between breaths helps reduce build‐up or helps prevent large swings in alveolar $P_{{\mathrm{CO}}_{2}}$ (Mortola & Limoges, 2006). However, in cetaceans the end‐tidal $P_{O_{2}}$ and $P_{{\mathrm{CO}}_{2}}$ are substantially lower and higher, respectively, and vary between breaths (Fahlman et al., 2015; Fahlman, Rhieu, et al., 2023). Based on the allometric regressions in Figure 2, the resting minute volume, the product of breathing frequency and tidal volume, is 24% higher in a 40 kg terrestrial mammal as compared with a cetacean, and this difference decreases to 15% for an animal weighing 5000 kg (Fahlman, Borque‐Espinosa, et al., 2020; Stahl, 1967). Assuming that the dead space ventilation is similar, this may explain the lower end‐tidal $P_{O_{2}}$ and higher $P_{{\mathrm{CO}}_{2}}$ in cetaceans as compared with terrestrial mammals.

In the horse during increasing levels of exercise, the breathing frequency increased by approximately 1900% (from ∼5 to ∼100 breaths min^−1^), while tidal volume only increased by 60% (from ∼5 to ∼8 litres) (Butler et al., 1993). As the exercise levels kept increasing, the respiratory demand in the horse was met by increasing tidal volume while breathing frequency remained more or less constant (Butler et al., 1993). In cetaceans, the vital capacity can be as high as 90% of TLC_est_, and studies using breathing frequency to estimate energy use have therefore assumed that the tidal volume is maximal, that is, equal to vital capacity, and increasing O_2_ demand to meet increasing metabolic requirements is provided by increasing breathing frequency (Fahlman et al., 2017, 2016). More recent studies indicate that the tidal volume of spontaneous breaths of resting marine mammals is not close to TLC_est_ or vital capacity (Fahlman, Borque‐Espinosa, et al., 2020). In cetaceans, data on tidal volume do not exist for exercise, but post‐exercise data in dolphins swimming against different drag loads showed that the tidal volume increased from about 30% of TLC_est_ during rest to about 55% of TLC_est_ for the first breath during recovery, but then rapidly declined exponentially back to the resting value (Fahlman et al., 2016). Similar results were seen in the same group of bottlenose dolphins following extended breath‐holds, where tidal volume and breathing frequency increased during the first initial breaths during recovery, although breathing frequency relatively more (Fahlman, Brodsky, et al., 2019).

In the cetacean, the expiratory volume of the relaxed lung is equal to the minimum air volume, and only about 7% of the TLC (Fahlman et al., 2017). Following a breath, the elastic recoil of the chest results in a lung that is almost free of air. In addition, the elastic recoil also helps generate high passive expiratory flow (see Figure 5a,b in Fahlman et al., 2017). When the elastic recoil is coupled with the respiratory intercostal muscles, the flow can be 10 times as high (around 160 litres s^−1^, or about 0.89 litres s^−1^ kg^−1^) as that measured in humans (see figure 5c in Fahlman et al., 2017). In the horse, the measured expiratory flow at rest was around 5 litres s^−1^ but increased up to 37 litres s^−1^ (about 0.08 litres s^−1^ kg^−1^) during maximal efforts (Butler et al., 1993). No measurements of respiratory flows has been made during exercise in cetaceans, but in the horse the expiratory flow increased to as high as 122 litres s^−1^ (0.27 litres s^−1^ kg^−1^) during maximal exercise. When expressed in vital capacities per second (VC s^−1^) the expiratory flow rates do not appear impressive in cetaceans (bottlenose dolphin: 6.1 VC s^−1^) and pinnipeds (California sea lion: 8 VC s^−1^) as compared with terrestrial mammals (human: 2 VC s^−1^; dog: 8 VC s^−1^; horse: 1–3.5 VC s^−1^; bat: 40 VC s^−1^) (Kooyman & Cornell, 1981; Leith, 1976). However, there is an inverse relationship between the flow expressed as VC s^−1^ and body mass (Leith, 1976), so if we compare the 450 kg horse with a 300 kg dolphin, the latter has a respiratory flow that is almost twice that of the horse.

**FIGURE 3:**
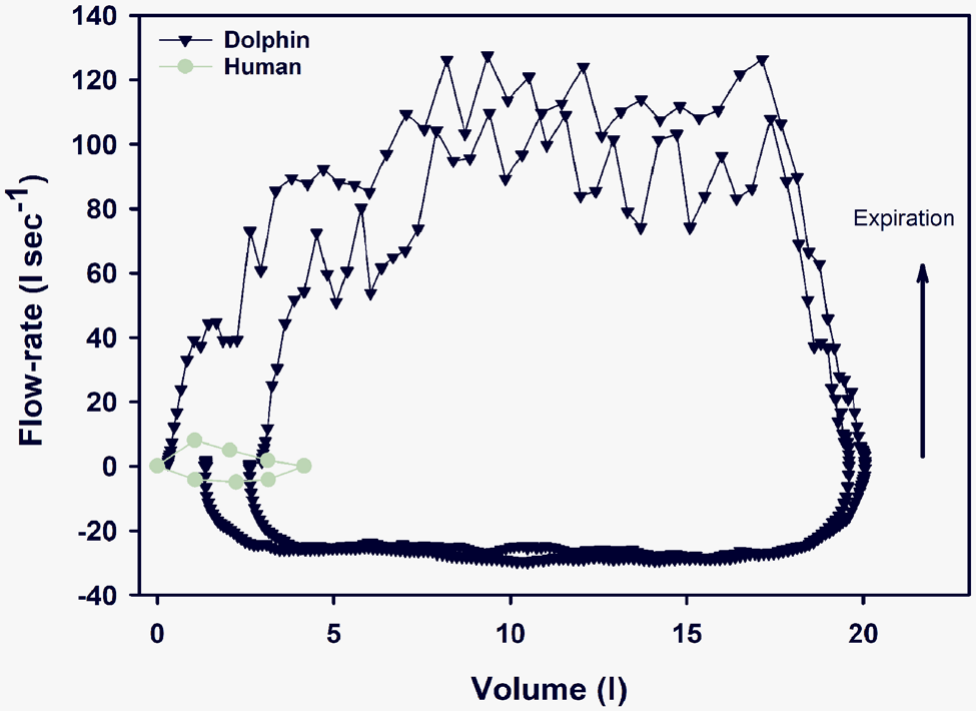
Flow–volume curves for two maximal breaths from a bottlenose dolphin and a single forced breath from a human. In the dolphin, the flow is effort dependent as indicated by the absence of changes in expiratory flow with changes in lung volume. Expiratory flow is positive, and exhalations have positive volume. Modified from Fahlman et al. (2015) and Bass (1973) with permission.

Maximal expiratory flow versus volume curves (flow–volume curves) provide another interesting comparison between marine and terrestrial mammals (Leith, 1976). In the human, and most other terrestrial mammals, the maximal expiratory flow reaches a peak at high lung volumes and decreases as the lung empties (Fahlman et al., 2017). Lung function data from excised lungs from the sei whale (*Balaenoptera borealis*), and harbour porpoise (*Phocoena phocoena*), and in trained California sea lions (*Zalophus californianus*), beluga whales (*Delphinapterus leucas*) and bottlenose dolphins (*Tursiops truncatus*) indicate that exhalation flow–volume curves are square formed (Figure 3). Thus, the anatomical design of the airways allows for fast tidal ventilation during the short surface period at times of travel when the animals spend brief periods at the water surface, such as porpoising.

The conducting airways in cetaceans, with complete cartilage rings, prevent tracheal compression during breaths and keeps the airways open (Moore et al., 2014). This architecture allows high expiratory flows throughout the breath (Figure 3) (Fahlman et al., 2018, 2017; Moore et al., 2014). In addition, these stiff conducting airways have been proposed to be the main adaptation that explains how marine mammals avoid excessive inert gas uptake and formation of gas emboli during ascent (Scholander, 1940). However, at the beginning of the century, necropsy studies of deep diving cetaceans reported gas emboli like those reported in diving humans with DCS (Bernaldo de Quirós et al., 2019; Hooker et al., 2012). After two decades of research, studies have shown that both marine mammals and turtles can experience diving‐related gas embolic pathology during periods of stress (Bernaldo de Quirós et al., 2019; Fahlman, Moore, et al., 2021; García‐Párraga et al., 2014; Hooker et al., 2012). Both empirical data and theoretical modelling indicated that despite stiff airways and a collapsible alveolar space, the repeated diving patterns seen in many airbreathing species indicate that the blood and tissue N_2_ tension should be high enough to regularly result in gas emboli during ascents (see Figure 3 in Fahlman, Moore, et al., 2021). Based on these modelling results, and from anatomical and physiological studies, the *selective gas exchange hypothesis* was formulated, which provided a physiological mechanism which explains how airbreathing diving species prevent uptake of N_2_ through adjustment of the ventilation and perfusion ratio (Fahlman, Moore, et al., 2021; García‐Párraga et al., 2018). This hypothesis stated that because of the large difference in gas solubility between O_2_, CO_2_ and N_2_, management of the ventilation perfusion ratio would allow some exchange of O_2_ and CO_2_ while minimizing or preventing exchange of N_2_ (Fahlman, Moore, et al., 2021; García‐Párraga et al., 2018).

**FIGURE 4:**
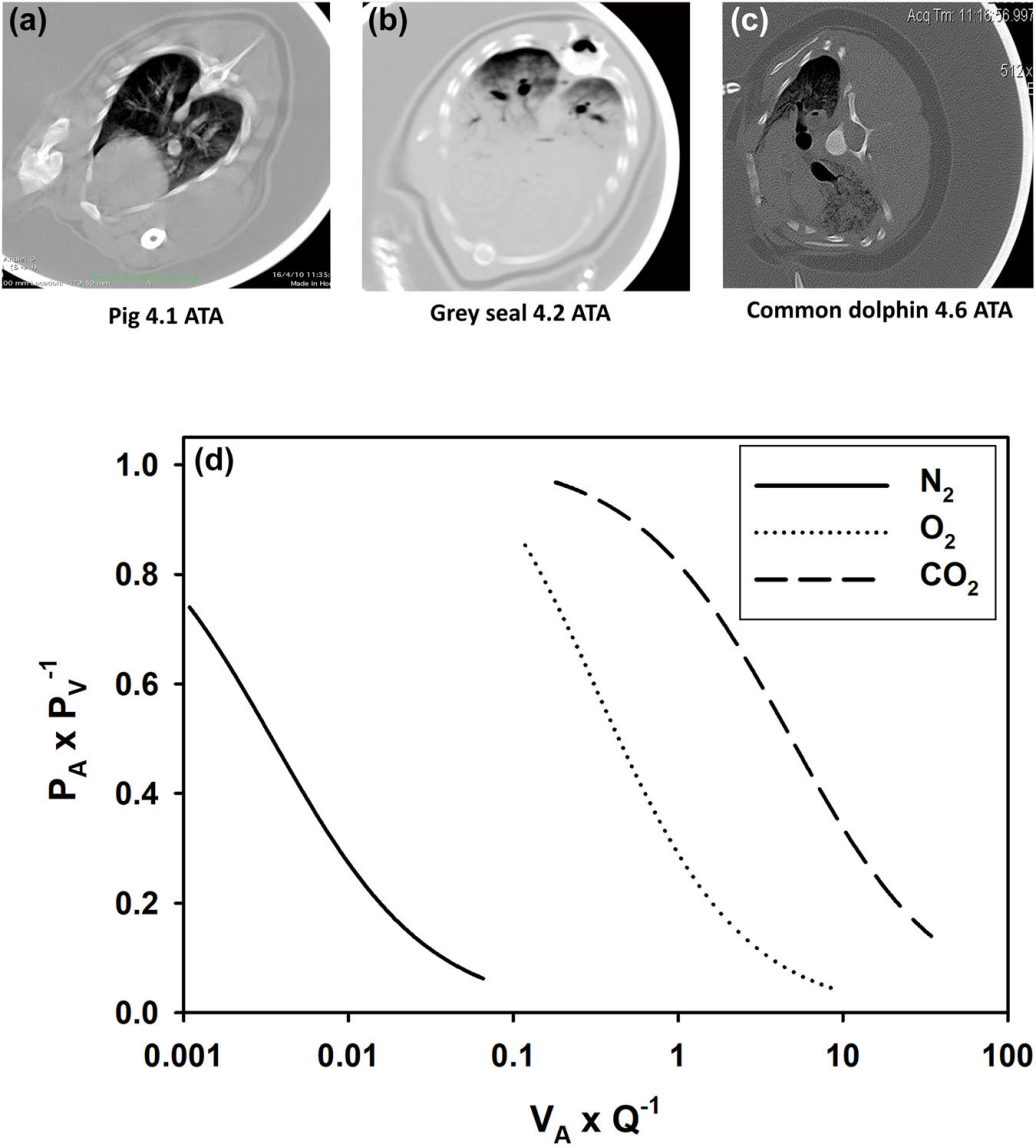
Hypothetical mechanism of the *selective gas exchange* hypothesis. (a–c) Computed tomographic images from pig (a), grey seal (b) and common dolphin (c) pressurized in a hyperbaric chamber to 4–4.5 ATA. These differences in pulmonary architecture result in pulmonary regions that are collapsed at shallow depths. (d) Altering the ventilation (${\dot{V}}_{A}$)/perfusion ($\dot{Q}$) ratio (${\dot{V}}_{A}x$ $\dot{Q}$ ^−1^) changes the alveolar (*P*_A_) and end‐capillary pulmonary venous (*P*_V_) tension for each gas. In a lung with increasing pulmonary shunt, the *P*_A_ *P*_V_^−1^ approaches 0, and in a lung with perfect matching the gas tensions are equal when leaving the lung, with the *P*_A_ *P*_V_^−1^ being 1. With a high ${\dot{V}}_{A}$ x $\dot{Q}$ ^−1^ ratio, exchange of O_2_ and CO_2_ is favoured, while a low ${\dot{V}}_{A}$ x $\dot{Q}$ ^−1^ ratio favours N_2_ exchange. Thus, conditioned capacity to vary heart rate, and hypoxic pulmonary vasodilatation allows cetaceans to vary the ventilation ${\dot{V}}_{A}$ x $\dot{Q}$ ^−1^ ratio, which allows O_2_ and CO_2_ exchange with minimal or no N_2_ exchange. Data and figures are reproduced with permission from Fahlman, Moore et al. (2021) and García‐Párraga et al. (2018).

In the dolphin, the *selective gas exchange hypothesis* assumes that the lungs, with excessive levels of collateral ventilation, compress heterogeneously (Fahlman, Moore, et al., 2021; García‐Párraga et al., 2018). This creates regions that are collapsed even at shallow depths. Evidence for this comes from hyperbaric computed tomography studies, where pulmonary compression results in alveolar regions that collapse unevenly in aquatic mammals, while in terrestrial mammals compression is homogeneous (Figure 4). The heterogeneous compression in aquatic mammals sets up two distinct regions, one collapsed and one open (Figure 4). Hypoxic pulmonary vasodilatation, which has been reported in sea lions (Olson et al., 2010), preferentially shunts gas to atelectic regions. This results in a high ventilation and perfusion ratio in ventilated regions, that is, high ventilation and minimal perfusion, which prevents or minimizes exchange of N_2_ and allows exchange of O_2_ and CO_2_ (Figure 4). The *selective gas exchange* hypothesis provides a mechanism that explains how cetaceans can avoid excessive uptake of N_2_ during diving. With a lung architecture that provides alveolar regions that are collapsed, and by matching pulmonary blood flow to the collapsed regions of the lung, the cetaceans can selectively exchange O_2_ and CO_2_ with little or no exchange of N_2_ (Fahlman, Moore, et al., 2021). The balloon‐pipe model proposed by Scholander (1940) relies entirely on passive compression, and neither empirical nor theoretical studies can explain how marine mammals are able to prevent gas emboli during ascent (see Figure 3 in Fahlman, Moore, et al., 2021). The *selective gas exchange hypothesis*, on the other hand, links anatomy and physiology, and explains how cetaceans are able to avoid N_2_ uptake during ascent. In this scenario, stress results in failure of ventilation–perfusion matching, which results in excessive inert gas uptake (see Figure 2 in Fahlman, Moore, et al., 2021).

## 2 THE DIVE RESPONSE

As early as 1870, Paul Bert provided early observations of cardiac changes during submersion in ducks (Bert, 1870). Later it was shown that this reduction in heart rate is also present in all vertebrates studied, and that breath‐hold diving resulted in bradycardia, peripheral vasoconstriction and reduced cardiac output (Scholander, 1940). These cardiovascular adjustments were proposed to be critical to conserve the available oxygen to organs like the brain and heart (Scholander, 1940). The extent of these cardiovascular changes has often been proposed to serve as one of the key adaptations that help to prolong a dive and an individual's and/or a species’ diving capacity (Kooyman, 1985). However, the cardiovascular change during submersion is also seen in species not thought to be adapted for diving. For example, in the human an extreme bradycardia of 6 beats min^−1^ was measured during face immersion (Arnold, 1985). It was therefore suggested that the ‘dive response’ is not an adaptation specific to diving, but an ancestral response to protect against hypoxia that has been conserved throughout phylogenetic history (Mottishaw et al., 1999). It has also been proposed that the cardiovascular responses during diving, in addition to having an O_2_ conserving effect, helps to assure that blood flow to the periphery and especially the muscle is reduced (Davis & Kanatous, 1999). In many marine mammals, the muscles have elevated levels of myoglobin, but due to the high O_2_ affinity of myoglobin the muscle $P_{O_{2}}$ has to decrease to allow the muscle to utilize this endogenous store (Davis & Kanatous, 1999; Fahlman et al., 2018; Fahlman, Allen, et al., 2023). Thus, a reduction in muscle blood flow assures that the muscle $P_{O_{2}}$ is reduced so that the endogenous O_2_ held by the myoglobin can be used by the muscle (Davis & Kanatous, 1999; Fahlman et al., 2018). This also conserves the haemoglobin‐bound O_2_ for organs like the brain and heart that have limited capacity for anaerobic metabolism.

**FIGURE 5:**
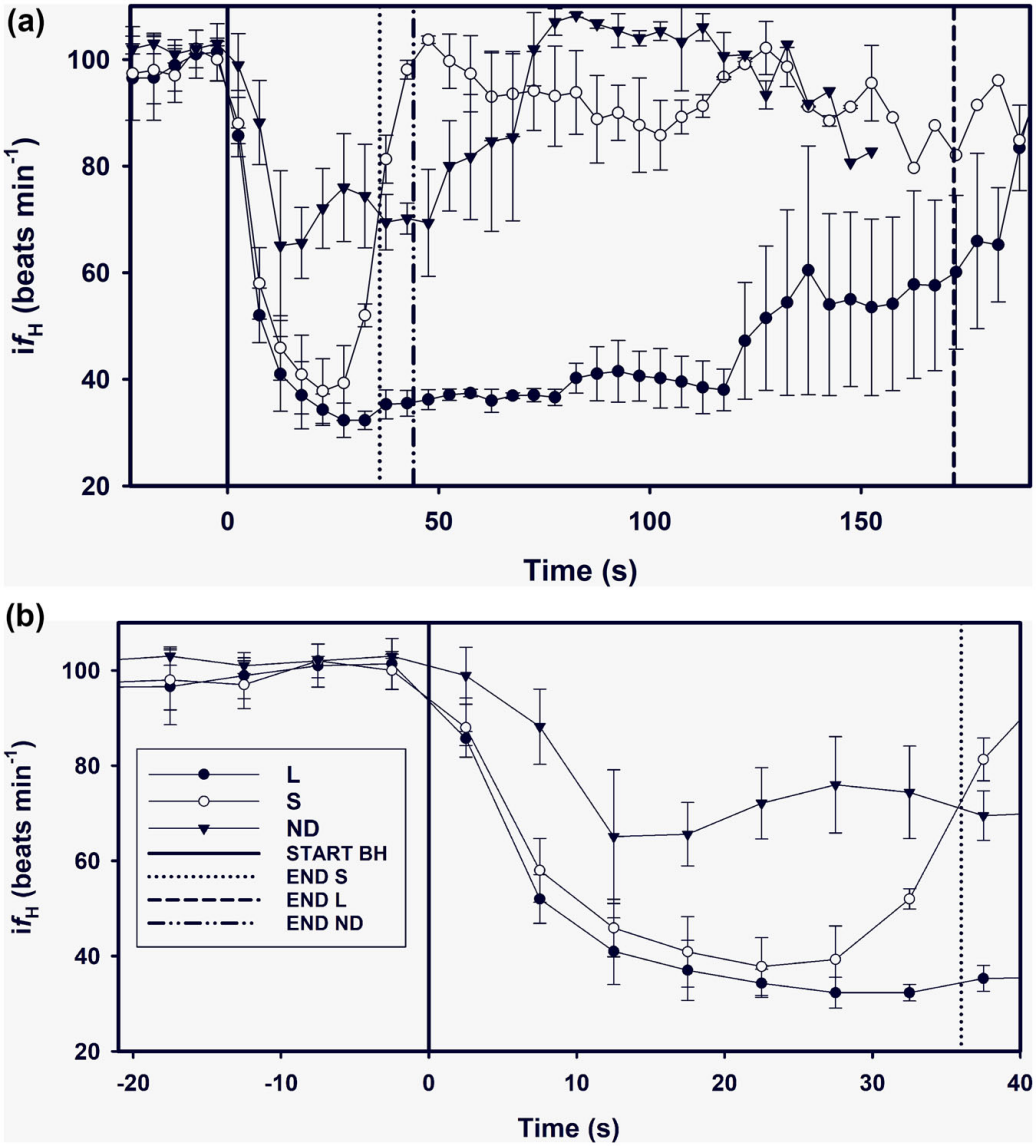
Instantaneous heart rate (i*f*_H_) against time before (time −20 to 0) and up to a) 193 s or b) 40 s following the start of the breath‐hold during three different types of breath‐holds (short (S), long (L) and no duration (ND)). During S and L breath‐holds, the dolphins were conditioned for a short or long breath‐hold using different signs. For the ND breath‐holds, the duration was determined by the dolphin without a prior signal. The continuous vertical black line is the start of the breath‐hold (BH) is when the dolphin took the last breath before submerging. The average time for the end of the breath‐hold and the time of the first breath after the breath‐hold are indicated as broken vertical lines for the SHORT, NS and LONG dives. Data are reproduced from Fahlman, Cozzi et al. (2020).

The reduction in heart rate during submersion is maintained by parasympathetic tone, while the peripheral vasoconstriction is governed by sympathetic tone (Ponganis et al., 2017). The rapid changes in heart rate at the initiation of the dive and as the dive ends is thought to be evidence that the diving heart rate is dominated by parasympathetic tone by the vagus nerve through what has been termed accentuated antagonism (Ponganis et al., 2017; Signore & Jones, 1995). Although the dive response has often been referred to as an autonomic reflex, numerous studies provide evidence that in marine mammals, at least, the response appears to be conditioned (Figure 5) (Elsner et al., 1966; Fahlman, Cozzi, et al., 2020; Ridgway et al., 1975). This allows diving animals flexibility in the response, where the diver can vary the magnitude of the dive response depending on the planned dive. Such a response may explain the short and shallow dives that often occur following deep and long dives, where elevated lung $P_{O_{2}}$ and maintained muscle perfusion help restore the muscle myoglobin stores (Fahlman et al., 2018; Fahlman, Allen, et al., 2023). Thus, variable cardiovascular changes in marine mammals suggest that the ‘dive response’ is not a pure autonomic reflex, but where dive‐adapted species have the capacity to vary both the temporal response and magnitude of the blood flow changes depending on the planned dive.

The cardiovascular changes associated with breathing, where both heart rate and stroke volume vary during the respiratory cycle, is another response reported in both marine and terrestrial mammals (Fahlman, Miedler, et al., 2019; Fahlman, Miedler, et al., 2020; Hirsch & Bishop, 1981; Ponganis, 2015; Ridgway, 1972). In both humans and dolphins, the magnitude of the heart rate changes has been shown to vary in relation to the breathing frequency and tidal volume (Blawas, Nowacek, Allen, et al., 2021; Blawas, Nowacek Nowacek, Rocho‐Levine, et al., 2021; Cauture et al., 2019; Hirsch & Bishop, 1981). There is some controversy in what direction the different breathing phases affect heart rate, but currently many studies propose that inspiration and expiration, respectively, cause acceleration and deceleration (Hirsch & Bishop, 1981; Looga, 1997). In addition, lung inflation has been shown to reverse bradycardia during apnoea, which highlights the influence that lung volume has on the heart rate during a breath‐hold (Angell‐James et al., 1981). In humans, this cardiac dependence on respiration has been proposed to enhance gas exchange through ventilation–perfusion matching or reducing the work of breathing while maintaining the $P_{{\mathrm{CO}}_{2}}$ isocapnic (Ben‐Tal et al., 2014). Therefore, the respiratory pattern (i.e., the breathing frequency, the tidal volume, and whether the breaths begin with an exhalation or inspiration) may alter the heart rate (Fahlman, Mcknight, et al., 2023).

In intermittent breathers, like the bottlenose dolphin, the instantaneous heart rate may double between breaths, ranging from 35–40 beats min^−1^ at the end of the inter‐breath period to between 80 and 90 beats min^−1^ after the breath (Blawas et al., 2021, 2021; Cauture et al., 2019; Ridgway, 1972). The same response is also reported in the killer whale (*Orcinus orca*), short‐finned pilot whale (*Globicephala scammony*), Pacific white‐sided dolphin (*Lagenorhynchus obliquidens*), Dall's porpoise (*Phocoenoides dalli*), common dolphin (*Delphinus* sp.), Amazon river dolphin (*Inia geoffrensis*) and the harbour porpoise (*Phocoena phocoena*) (Blawas et al., 2021b; Kanwisher & Sundness, 1965; Ridgway, 1972). In the bottlenose dolphin, for example, with a forceful exhalation ranging between 30 and 50 litres s^−1^ during calm breaths to 100 and 160 litres s^−1^ during forceful breaths, and an immediate inhalation, the instantaneous heart rate reaches its maximal value around 7–12 s following the end of the inhalation, but in some circumstances it can take as long as 20 s (Cauture et al., 2019; Fahlman, Miedler, et al., 2019; Fahlman, Miedler, et al., 2020; Ridgway, 1972). The variation in the time course to reach the maximal instantaneous heart rate may be due to the opposite effects of the exhalation and inhalation (Fahlman, Mcknight, et al., 2023). These opposite effects of heart rate during the respiratory phase may be caused by the pulmonary (variation in lung volume) or atrial (variation in venous return) stretch receptors (Ponganis et al., 2017). Thus, the tight coupling between breathing and heart rate should be considered and accounted for when comparing cardiac function within and between species. These and previous data presented suggest that other ways to analyse heart rate, such as median heart rate or the ratio of heart rate and breathing frequency, should be considered to achieve better comparisons within different conditions for the same species or between different species.

Heart rate is often assumed to correlate with cardiac output, and thereby provide an estimate of the oxygen delivery and aerobic demand during a dive (Kooyman, 1985). The diving heart rate is compared with the heart rate at the surface, for example, resting heart rate (Noren et al., 2012), where the latter provides an estimate of the circulatory requirements without limitations to oxygen. The close coupling between breathing and cardiac function, therefore, makes it challenging to estimate a true resting heart rate, or a heart rate that provides comparative value, especially in intermittent breathers. Using the resting/normal heart rate as a baseline index for cardiovascular function to meet resting or basal metabolic needs, is heavily influenced by the respiratory frequency (Blawas, Nowacek, Allen, et al., 2021; Blawas, Nowacek Nowacek, Rocho‐Levine, et al., 2021; Fahlman, Miedler, et al., 2020). Because of this, it was suggested that the heart rate during diving should be compared against the resting heart rate during apneusis (Kooyman, 1985). Such a comparison in the bottlenose dolphins shows that the heart rate following a 15–20 s inter‐breath interval is similar to those following a 240 s submerged breath‐hold (Blawas, Nowacek, Allen, et al., 2021; Blawas, Nowacek Nowacek, Rocho‐Levine, et al., 2021; Fahlman, Miedler, et al., 2019; Fahlman, Miedler, et al., 2020; Noren et al., 2012). Similar results have been reported in other mammals, and in birds and reptiles (Belkin, 1964; Gaunt & Gans, 1969; Kanwisher et al., 1981). Based on data from these species, it was shown that when accounting for the confounding effects of breathing there was no diving bradycardia (Kooyman, 1985; Kooyman & Campbell, 1972; Lin et al., 1972). In the freshwater river cooter (*Pseudemys concinna*), it was reported that during dives within the aerobic dive duration, the observed changes in heart rate were a tachycardia associated with breathing and not a bradycardia caused by diving (Belkin, 1964). Similar results were reported in the Weddell seal (*Leptonychotes weddellii*), where there was little or no difference in the heart rate during the apneustic period while resting at the surface as compared with during dives shorter than 10 min (Kooyman & Campbell, 1972). During longer dives (>10 min), on the other hand, a diving bradycardia was reported which was highly variable but varied with dive duration (Kooyman & Campbell, 1972). In the bottlenose dolphin, it was also seen that the heart rate during breath‐holds up to 5 min was similar to those during the inter‐breath interval (Fahlman, Miedler, et al., 2019). Thus, it appears that both in reptiles and in mammals, the heart rate during dives within the aerobic dive limit is not different from that between breaths. During short dives, the diving heart rate is seldom below that seen between breaths, but can be modulated by dive duration, activity during submersion or anticipation (Elsner et al., 1966; Fahlman, Miedler, et al., 2019; Fahlman, Cozzi, et al., 2020; Kooyman, 1985; Ponganis et al., 2017). The diving heart rate varies with dive duration in seals and dolphins both during active and static breath‐holds (Fahlman, Miedler, et al., 2019; Kooyman & Campbell, 1972; Ridgway et al., 1975). Although it has been proposed that pressure (depth) alters the diving heart rate, possibly through pulmonary stretch receptors (Ponganis et al., 2017), the confounding effect with activity, diving lung volume and the positive relationship between dive depth and dive duration makes it difficult to assess this proposed effect of depth. For example, in trained freely diving bottlenose dolphins, there was no effect of the average diving heart rate with depth (Houser et al., 2010). It is possible that deeper and/or longer dives begin with a higher diving lung volume, as has been shown in the California sea lion (McDonald & Ponganis, 2012), which may further confound such a relationship. In the harbour seal and human, exhalation and inspiration, respectively, result in cardiac deceleration and acceleration, and the magnitude of the latter is affected by tidal volume (Angell‐James et al., 1981). In the bottlenose dolphin, both exhalation and inhalation alter heart rate, with the latter resulting in acceleration (Fahlman, Mcknight, et al., 2023). Therefore, the diving heart rate is also likely affected by the diving lung volume, and depth per se may not be the important factor that alters diving heart rate. The evidence that the variation in diving heart rate can be conditioned may also influence the results in previous studies, where it has been shown that the overall magnitude of the dive response, and also the rate of heart rate reduction at the beginning of the dive may be altered by anticipation of the dive (Figure 5) (Elsner et al., 1966; Fahlman, Cozzi, et al., 2020; Ridgway et al., 1975). Thus, there is a need to carefully assess the many potentially factors that may alter cardiac function during diving in controlled studies or statistically separate the various confounding factors in studies on wild animals to confirm results and conclusions.

## 3 SUMMARY AND CONCLUSION

A better understanding of the adaptations that allow breath‐hold diving animals to perform long and deep dives may help with conserving these species from the threats of climate change, increasing ocean noise, pollution and over‐exploitation of marine resources. In addition, these adaptations may also provide novel treatments for human diseases (He et al., 2023; Ponganis, 2019). For example, atelectasis is a common problem in human emergency medicine or during prolonged surgery, where a positive pulmonary pressure in the intubated patient is often used to keep the alveoli open, which may result in alveolar rupture. Both pinnipeds and cetaceans appear to have lung surfactants that allow the alveoli to open more easily (Foot et al., 2007; Gutierrez et al., 2015; Miller et al., 2006). Improved understanding of the cardiorespiratory coupling may help better understand how marine mammals avoid injury from hypoxia and ischaemia–reperfusion. Thus, future studies to define the physiological capacity of marine mammals will not only help with conservation efforts but may also have benefit for humans. Emerging technologies, such as near infrared spectroscopy (McKnight et al., 2019) and electrical impedance tomography (Adler et al., 2021), applied to aquatic mammals will provide novel studies in physio‐logging (Fahlman, Aoki, et al., 2021) that are likely to revolutionize the field of marine mammal physiology. These will be able to confirm the many hypotheses postulated by Scholander (1940) in his seminal study.

## AUTHOR CONTRIBUTIONS

All authors have approved the final version of the manuscript and agree to be accountable for all aspects of the work. All persons designated as authors qualify for authorship, and all those who qualify for authorship are listed.

## CONFLICT OF INTEREST

The author declares no conflict of interest.
